# Synthesis and Modification of Nanostructured Thin Films

**DOI:** 10.3390/nano9101427

**Published:** 2019-10-09

**Authors:** Ion N. Mihailescu

**Affiliations:** Lasers Department, Laser-Surface-Plasma Interactions Laboratory, National Institute for Lasers, Plasma, and Radiation Physics (INFLPR), Strada Atomistilor, nr. 409, P.O. Box MG-36, RO-077125 Magurele, Ilfov, Romania; ion.mihailescu@inflpr.ro

The idea of nanomaterials, nanoscience, and nanotechnologies was formulated by Richard Feynman in 1959 in his famous lecture “There’s Plenty of Room at the Bottom”. He said that “The principles of physics, as far as I can see, do not speak against the possibility of maneuvering things atom by atom”. 

Since then, a nanomaterials “revolution” followed, confirming their superior properties, as e.g., toughness, strength, hardness, resistance to corrosion and wear, and various thermal, magnetic, and optical features. In this context, the fabrication and characterization of thin films remains the main cornerstone of nanotechnologies. 

As a rule, films are deposited onto solid surfaces to obtain better properties. A thin film is basically defined as a low-dimension material fabricated by assembling atomic/molecular/ionic species with a final thickness in the nm range. Nano-thin films can be essentially synthesized from any kind of material, which opens the way to vast application domains. 

This Special Issue on “Synthesis and Modification of Nanostructured Thin Films” contains contributions about thin film synthesis, modification, and characterization for potential applications in leading domains. This collection of 18 research papers represents 136 authors from 12 countries, and is devoted to advanced topics in both the synthesis (13) as well as the modification (5) of nanostructured thin films. In particular, the thickness of films ranges from a few up to 250 nm.

The major compounds of key interest were studied, including AlGaN [1], Cu NWs [2], photonic crystal fibers [3], LiNbO_3_ [4], Au nanoparticles [5], Al_2_O_3_/Tm_2_O_3_ [6], Ge-DLC [7], Mo/Ti [8], ZnTe:Cu [9], Ge–Sb–Te [10], noble metal nanoparticles [11], collagen/Zn^2+^-substituted calcium phosphates [12], TiO_2_ [13], Si-DLC [14], SHG in ZnO nanofilms [15], Cu_2_Mg_x_Zn_1−x_SnS_4_ [16], ethylene vinyl acetate (EVA) matrices [17], and LIPSS [18].

In the opinion of this Editor, the main characteristic of this selection is the quite large range of applications, which extends from nanobiomedicine to solar cells. 

Finally, one should stress upon the original character of all contributions, which will serve as active vectors in the years to come for the further dynamic development of new nanostructured thin film systems. 

## References

[1] Tasi C.-T., Wang W.-K., Ou S.-L., Hunag Y.-S., Horng R.-H., Wuu D.-S. (2018). Structural and Stress Properties of AlGaN Epilayers Grown on AlN-Nanopatterned Sapphire Templates by Hydride Vapor Phase Epitaxy. Nanomaterials.

[2] Mock J., Bobinger M., Bogner C., Luigi P., Becherer M. (2018). Aqueous Synthesis, Degradation, and Encapsulation of Copper Nanowires for Transparent Electrodes. Nanomaterials.

[3] Malka D., Katz G. (2018). An Eight-Channel C-Band Demux Based on Multicore Photonic Crystal Fiber. Nanomaterials.

[4] Wu R., Wang M., Xu J., Qi J., Chu W., Fang Z., Zhang J., Zhou J., Qiao L., Chai Z. (2018). Long Low-Loss-Litium Niobate on Insulator Waveguides with Sub-Nanometer Surface Roughness. Nanomaterials.

[5] Rout A., Boltaev G.S., Ganeev R.A., Fu Y., Maurya S.K., Kim V.V., Srinivasa K.R., Guo C. (2019). Nonlinear Optical Studies of Gold Nanoparticle Films. Nanomaterials.

[6] Liu Y., Ouyang Z., Yang L., Yang Y., Sun J. (2019). Blue Electroluminescent Al_2_O_3_/Tm_2_O_3_ Nanolaminate Films Fabricated by Atomic Layer Deposition on Silicon. Nanomaterials.

[7] Jelinek M., Kocourek T., Jurek K., Jelinek M., Smolková B., Uzhytchak M., Lunov O. (2019). Preliminary Study of Ge-DLC Nanocomposite Biomaterials Prepared by Laser Codeposition. Nanomaterials.

[8] Shen H., Yao B., Zhang J., Zhu X., Xiang X., Zhou X., Zu X. (2019). Effect of Thickness of Molybdenum Nano-Interlayer on Cohesion between Molybdenum/Titanium Multilayer Film and Silicon Substrate. Nanomaterials.

[9] Chen B., Liu J., Cai Z., Xu A., Liu X., Rong Z., Qin D., Xu W., Hou L., Liang Q. (2019). The Effects of ZnTe:Cu Back Contact on the Performance of CdTe Nanocrystal Solar Cells with Inverted Structure. Nanomaterials.

[10] Bulai G., Pompilian O., Gurlui S., Nemec P., Nazabal V., Cimpoesu N., Chazallon B., Focsa C. (2019). Ge-Sb-Te Chalcogenide Thin Films Deposited by Nanosecond, Picosecond, and Femtosecond Laser Ablation. Nanomaterials.

[11] Tommasini M., Zanchi C., Lucotti A., Bombelli A., Villa N.S., Casazza M., Ciusani E., de Grazia U., Santoro M., Fazio E. (2019). Laser-Synthesized SERS Substrates as Sensors toward Therapeutic Drug Monitoring. Nanomaterials.

[12] Neacsu I.A., Arsenie L.V., Trusca R., Ardelean I.L., Mihailescu N., Mihailescu I.N., Ristoscu C., Bleotu C., Ficai A., Andronescu E. (2019). Biomimetic Collagen/Zn^2+^-Substituted Calcium Phosphate Composite Coatings on Titanium Substrates as Prospective Bioactive Layer for Implants: A Comparative Study Spin Coating vs. MAPLE. Nanomaterials.

[13] Lungu J., Socol G., Stan G.E., Ştefan N., Luculescu C., Georgescu A., Popescu-Pelin G., Prodan G., Gîrţu M.A., Mihăilescu I.N. (2019). Pulsed Laser Fabrication of TiO_2_ Buffer Layers for Dye Sensitized Solar Cells. Nanomaterials.

[14] Bociaga D., Sobczyk-Guzenda A., Komorowski P., Balcerzak J., Jastrzebski K., Przybyszewska K., Kaczmarek A. (2019). Surface Characteristics and Biological Evaluation of Si-DLC Coatings Fabricated Using Magnetron Sputtering Method on Ti6Al7Nb Substrate. Nanomaterials.

[15] Long H., Habeeb A.A., Kinyua D.M., Wang K., Wang B., Lu P. (2019). Influences of Ga Doping on Crystal Structure and Polarimetric Pattern of SHG in ZnO Nanofilms. Nanomaterials.

[16] Sui Y., Zhang Y., Jiang D., He W., Wang Z., Wang F., Yao B., Lili Y. (2019). Investigation of Optimum Mg Doping Content and Annealing Parameters of Cu_2_Mg_x_Zn_1−x_SnS_4_ Thin Films for Solar Cells. Nanomaterials.

[17] Mariotti G., Vannozzi L. (2019). Fabrication, Characterization, and Properties of Poly (Ethylene-Co-Vinyl Acetate) Composite Thin Films Doped with Piezoelectric Nanofillers. Nanomaterials.

[18] Alves-Lopes I., Almeida A., Oliveira V., Vilar R. (2019). Influence of Femtosecond Laser Surface Nanotexturing on the Friction Behavior of Silicon Sliding Against PTFE. Nanomaterials.

